# Perceptions and attitude of women of Luderitz, Namibia on Pap smear and cervical cancer prevention

**DOI:** 10.1186/s12905-022-01698-x

**Published:** 2022-04-21

**Authors:** Lucia Hausiku, Koffi Kouame, Yapo Guillaume Aboua

**Affiliations:** 1 Department of Health Sciences, Faculty of Health and Applied Sciences, Namibia University of Science and Technology, Private Bag 13388, Windhoek, 9000 Namibia; 2 Department of Human Biology, Faculty of Health Sciences, Walter Sisulu University, P O Box PBag×1 Nelson Mandela Drive Campus, Mthatha, 5099 South Africa

**Keywords:** Attitude, Carcinoma, Cervical cancer, Pap smear, Perceptions, Women

## Abstract

**Background:**

Cervical cancer is one of the leading malignancies globally and has taken third place in Namibia amongst women aged 15–44 years. Infection with the Human Immunodeficiency Virus (HIV) has been proven to increase women’s susceptibility to developing cervical carcinoma. Sadly, Namibia carries a twin burden of HIV and cervical cancer. Namibians are aware of HIV/AIDS, but remain poorly informed about cervical cancer. Furthermore, among those who are aware of the disease, low utilisation of screening tests have been reported.

**Objective:**

The purpose was to explore perceptions and attitudes held by women about cervical cancer, reasons for low uptake of Pap smear testing amongst those who are aware of the malignancy as well as unearth motivation factors that has fuelled women to go for screening.

**Methodology:**

A descriptive, cross-sectional study was conducted using convenience sampling as a sampling technique. The survey instrument used was a self-administered questionnaire. It consisted of both closed and open ended questions. A total of 136 women were surveyed.

**Results and conclusion:**

The level of awareness for cervical cancer (92.6%) and Pap smear (93.4%) were high. Most were able to identify that Pap smear test is used for screening for pre-cancerous lesions. However, knowledge about the impact of a HIV positive status along with co infection with HPV as the leading causes for progression of invasive cervical carcinoma was not well known. Knowledge about the other risk factors such as multiple sexual partners (39.7%), early sex debut (34.9%) and smoking was poorly demonstrated. This suggests that a high awareness level does not necessarily translate into having a good perception or understanding of a disease. A good attitude towards screening was observed although less than half of the study population reported ever having a test done.

## Introduction

Cervical cancer screening uptake remains low in developing countries because of lack of resources and expertise [1]. In addition, prevalence of cervical cancer in Sub-Saharan Africa, still at high level due to competing health needs as most of these countries still lack services for the prevention, such as early diagnosis and management [2].

The reduction in the prevalence cervical cancer is due national screening programmes in many developed nations, while developing nations lack resources and mainly practice sporadic screening. Cervical cancer is preventable if detected early [3]. The Ministry of Health and Social Services (MoHSS) of Namibia adopted that Papanicolou (Pap) smear be done to sexually active, and HIV infected women aged from 15 and 49 years.

In addition, the Guidelines for Management of Sexually Transmitted Infections, using the Syndromic Approach advocates that all eligible pregnant women visiting the health facilities be screened as part of medical physical examination [4] and that all postnatal women be screened for cervical cancer six weeks after delivery [5].

Cervical cancer can be prevented by early detection through use of one of the selected methods such as Pap smear, Visible Inspection with Acetic (VIA), Visual Inspection using Lugol’s Iodine (VILI) and acid Human Papilloma virus Deoxyribonucleic acid (HPV DNA) testing identified as the most important cervical cancer preventing strategy [6]. However, not all these methods are readily available in sub-Sahara due to challenges of health infrastructures.

Pap smear is a procedure where cells from the cervix are collected and studied under a microscope to detect pre-cancer and cancer cells with the aim to detect early and prevent cervical cancer [7]. About 80% of cervical cancer cases can be managed and reduced through early detection via Pap smear [8]. Pap smear (cytology) test has been used in large population and led to a reduction of cervical cancer incidence and mortality [9]. However, sustaining high-quality cytology-based programmes is impossible in low-resource settings and therefore the most efficient and effective strategy for detecting and treating cervical cancer precursors in low-resource settings remains to screen using either VIA, VILI or HPV DNA testing and then to treat using cryotherapy (freezing) [9]. In addition there is uncertainty of women coming back for Pap smear results [10]. Studies reveal that specificity and sensitivity of VIA and Pap smear are largely comparable [11]. HPV DNA testing has been identified as the most important cervical cancer preventing strategy [6]. VIA and VILI may become an alternative screening tool as they are simple, easy, rapid to administer and do not need many instructions and highly specialized medical staff, are cost effective and results can be made available immediately [9].

Access to information and knowledge influences health decisions making. Information, knowledge and access to health facilities influence women’s decision making for screening for cervical cancer. According the NDHS only 25% of Namibian women aged 15–49 years were screened for cervical cancer in 2013 despite the availability of a national cancer screening programme [12]. Namibia carries a twin burden of cervical cancer and HIV [13]. The prevalence of HIV/AIDS in Luderitz was reported to be 20.9% for the year 2014. This figure was significantly higher than the national average which stood at 16.9% [14]. According to the cancer registry, 39 cervical cancer cases have been reported in the! Karas region between 2010 and 2016. For a highly preventable, non-communicable disease, these figures are worrisome. HIV/AIDS is known to increase women’s susceptibility to cervical cancer [15]. The rise in HIV/AIDS incidence may lead to an increased number of cervical cancer cases the town of Luderitz. Cervical cancer rates are highest in Eastern Africa including Namibia while minimal in developed countries due to well-organized screening programs and HPV vaccination of girls from the ages of 9 to 13 to prevent infection caused by HPV, which reduce the overall risk of cervical cancer. The World Health Organization (WHO) estimates that at least one third of all HPV-related cancers in Africa could be prevented with comprehensive vaccination implementation [1]. In 2019 doses of HPV vaccine in females in Namibia was unknown. This study has been conducted to assess the perceptions, knowledge and attitudes of women undergoing cervical cancer screening in Luderitz, Namibia.

## Methods

### Study design

This was a non-probability sampling method (Convenience Sampling) descriptive, cross-sectional, quantitative study to assess knowledge, perceptions and attitudes to determine their frequency. A questionnaire consisted of both closed and open-ended questions to gain a deeper understanding into the targeted population’s attitudes and perceived beliefs of the disease was distributed to participants. Reliability test was done to examine whether the questions were interpreted amongst the women in a similar fashion to allow changes to be made if such disparities were noted. It was found that the questions were all understood similarly, and no misinterpretations were observed during this phase, therefore making it reliable enough in its ability to elicit the right responses. Participants in the study were selected among females of all ethnic groups who have been living and residing in Luderitz for five years or more and of age between 21 and 60.

### Sample size

$${\text{n}} = ({\text{Z}}\upalpha /{2})^{{2}} {\text{P(1}} - {\text{P)}}/{\text{d}}^{{2}}$$

Z = 95% confidence interval (1.96)

d = Marginal error = 8%

n = sample size

P = estimated proportion 66%

Zα/2 = Critical value [16]

For survey type research, the above formula is used to estimate sample size. According to a Namibian study, the awareness of cervical cancer was 66% [17]. This study therefore used p = 66%, an error margin of 8% with a confidence interval of 95% to calculate the sample size. The sample size calculated was 158.466 ≈159. A non-response rate of 30% was factored in, making the adjusted sample size 207. Due to time constraints, incomplete and unreturned questionnaires from the targeted participants, a total of 136 completely filled questionnaires were used in the study.

### Ethical considerations

Ethical clearance was sought from the Research Ethics Screening Committees of the Namibia University of Science and Technology (NUST, ethical number: FHAS 15/2019) as well as the Ministry of Health and Social services (Ethical clearance number LH 2019) to conduct the study. Thereafter, individuals were approached and allowed to participate in the study based on their own free will and anonymity and confidentiality were observed. All methods were performed in accordance with the relevant guidelines and regulations.

## Results

Sociodemographic variables and perception of participants on Pap smear.

A total of 136 women volunteered were selected to be part of the study. The majority (28.7%) of them fell within the age group of 41–60 years, with Christianity (97.8%) being the predominant religion (Table 1). Close to a third of the sample, n = 48 (35.3%) had senior secondary education, while junior and tertiary education stood at 29.4% and 28.7% respectively. Only one individual reported having primary education (0.7%). Most of the women were single n = 98 (72.1%), while n = 34 (25%) are married. Out of the 136 participants, 32 (23.5%) have no child, 32 (23.5%) have 1 child, 70 (51.5%) have 2 to 5 children and 2 (1.4%) have more than 5 children. Majority of the participants 119 (87.5%) were employed while 13 (9.6%) of the participants were without employment.

**Table 1:** Sociodemographic variables of participants n = 136

	Frequency	%
Age group		
21–25	24	17.6
26–30	28	20.6
31–35	31	22.8
36–40	14	10.3
41–60	39	28.7
Total	136	100.0
Religion		
Christian	133	97.8
Muslim	0	0.0
Traditionalist	0	0.0
No religion	3	2.2
Total	136	100.0
Education level		
No formal education	1	0.7
Primary education	8	5.9
Junior secondary	40	29.4
Senior secondary	48	35.3
Tertiary education	39	28.7
Employment status		
Employed	119	87.5
Unemployed	13	9.6
Self employed	4	2.9
Retired	0	0.0
Marital status		
Single	98	72.1
Married	34	25.0
Divorced	1	0.7
Widowed	2	1.5
Engaged	1	0.7
Number of children		
0	32	23.5
1	32	23.5
2	37	27.2
3	26	19.1
4	5	3.7
5	2	1.5
6	1	0.7
7	1	0.7

According to Table 2 all women aged ‘36- 60’ have heard of cervical cancer and Pap smear. Although only 81% of women in the age group ‘31–35’ said they have heard about cervical cancer, 84% stated that they have heard about Pap smear testing. This gives a picture that there are some women who are aware of Pap smear testing but not aware of cervical cancer. Out of 24 women aged ‘21–25’, n = 23 (96%) of them stated that they have heard of Cervical cancer and Pap smear.

**Table 2:** Proportion of women of Luderitz who have heard about Pap smear by age group

Number of participants who heard about Pap smear by age group				
Age group	Heard about Pap smear		Total number of participants	Proportion in %
	No	Yes		
21–25	1	23	24	96%
26–30	3	25	28	89%
31–35	5	26	31	84%
36–40		14	14	100%
41–60		39	39	100%
Grand total	9	127	136	93.4

### Perception of Cervical cancer, risk factors and prevention methods

A large portion of women stated having heard of cervical cancer (92.6%) and Pap smear test (93.4%) (Table 3). However, when asked what the Pap smear test is used for, a variety of responses were chosen. Most agreed that it’s to screen for cervical cancer n = 111 (87.4%). Other responses were; treating sexual transmitted disease n = 6 (4.7%), screening for infertility n = 6 (4.7%), womb cleaning n = 9 (7.1%) and bladder cancer n = 1 (0.8%) (Table 4).

**Table 3:** Perception of Cervical cancer, Pap smear, risk factors, and prevention amongst women

Variables	Responses	Frequency	%
Use of Pap smear (multiple responses)	To treat sexual transmitted disease	6	4.7
	To screen for cervical cancer	111	87.4
	Cleaning of the womb	9	7.1
	Removal of womb	3	2.4
	To screen for infertility	6	4.7
	Don't know	4	3.1
	Other use*	1	0.8
Total		127	100.0
Risk factors (multiple responses)	Single response	45	35.7
	Two responses	109	86.5
	One of the correct responses	59	46.8
	Two correct responses	10	7.9
Do you think you are at risk	Yes	72	52.9
	No	54	39.7
	Don’t know	10	7.4
Prevention methods (multiple responses)	Avoiding multiple sexual partners	61	48.4
	Avoiding early sex debut	32	25.4
	Quit smoking	33	26.2
	Regular screening	94	74.6
	HPV vaccination	21	16.7
	Non oral contraceptive	5	4.0
	Other prevention methods**	7	5.6
Treatment available for cervical cancer	Yes	107	84.9
	No	14	11.1
	Don't know	5	4.0
Total		126	100.0

*Other use included bladder cancer (0.8%)

** Others included don’t use soap (1.6%), Use protection during intercourse (2.4%), using clean toilets (1.6%)

**Table 4:** Pap smear screening uptake amongst participants according to age group n = 63

Age group	Screening count by age group		
	Frequency	Total number of participants	%
21–25	6	24	25
26–30	10	28	36
31–35	12	31	39
36–40	10	14	71
41–60	25	39	64
Grand total	63	136	100

When participants were asked to state two factors that carry the greatest risk for cervical cancer development. About one third gave a single response only n = 45 (35.7%). Those who gave sufficient responses (two answers) comprised of 109 women (86.5%). Those who mentioned one of the two correct responses consisted of 59 women (46.8%). Only n = 10 (7.9%) participants correctly identified that having a weak immune system caused by HIV along with being infected with HPV are the highest risk factors toward cervical cancer development (Table 4). The risk factors frequently stated were; having multiple sexual partners n = 50 (39.7%), early sexual debut n = 44 (34.9%), smoking n = 22 (17.5%) and long term oral contraceptive use n = 20 (15.9%). Other risk factors included alcohol use (0.8%), dirty toilets (1.6%), lack of regular screening (0.8%), Microwaved food (0.8%), Soap (1.6%), Spicy food (0.8%), Unprotected sex (0.8%), Genetics (0.8%), High blood pressure (0.8%), Lotions (0.8%), Plastics (0.8%). Irregular menstruation (0.8%), was reported as a risk factor, when in fact, it’s a symptom of cervical carcinoma.

When asked about whether they thought they are at risk of developing cervical cancer. Over half indicated that they were at risk of developing cervical cancer n = 72 (52.9%), while n = 54 (39.7%) did not think they were. Others stated that they did not know whether they were at risk n = 10 (7.4%) (Table 3).

About three quarters of the sample n = 94 (74.6%) stated regular screening as a preventive measure. Close to half n = 61 (48.4%) stated that cancer of the cervix can be prevented through avoiding multiple sexual partners, avoiding early sex debut n = 32 (25.4%) and quitting smoking n = 33 (26.2%). Only n = 21 (16.7%) stated HPV vaccination. A smaller proportion stated not using soap to wash the vagina (1.6%) and making use of clean toilets (1.6%) as measures to prevent cervical carcinoma (Table 4). No one was able to mention all the preventive measures for cervical cancer.

A greater proportion reported that cervical carcinoma can be treated n = 107 (84.9%), n = 14 (11.1%) said there was no treatment and n = 5 (4.0%) did not know whether treatment was available (Table 3).

Table 4 indicate that among the total participants who took part in the study n = 136, less than half were screened n = 63 (46.3%), majority of which were women between the ages of 36–40 (71%), followed by age group 41- 60. Amongst the n = 24 women aged 21–25, only 6 went for screening (25%).

According to Table 3 illustrated below, their reasons for screening was mostly due to doctor recommendation (29%), wanting to be informed about their health status/wellbeing (24%) and knowing someone who previously got screened (21%). Others got screened as part of antenatal (2%), and post-natal procedures (3%). Some women stated that they had worrying symptoms which led to them going for screening (13%).

According to Table 5, most women have gone for screening on more than two occasions n = 28 (46.3%). When asked where they got their information from about the test, majority stated that they were informed by their healthcare workers n = 22 (36%), followed by relative/friends n = 11 (18%). Others stated Pamphlets n = 10 (16%), Posters n = 6 (10%). Only n = 1 (2%) stated school being their source of information.

**Table 5:** Motivation, frequency for screening amongst women n = 63

Motivations for screening		Frequency	%
What motivated you to get screened? (multiple response applicable)	Doctor recommendation	18	29
	Had worrying symptoms	8	13
	know of someone who got screened	13	21
	Antenatal screen	1	2
	awareness of dangers of cervical cancer	1	2
	Colleague recommended	1	2
	Fibroids	1	2
	For prevention purpose	1	2
	postnatal testing	2	3
	Relative recommended	1	2
	Routine testing	2	3
	To be informed about health/well-being	15	24
How many times	Once	23	38
	Twice	12	20
	more than two times	28	46
How did you find out about Pap smear test?*	Pamphlets	10	16
	Relative/friend	11	18
	Healthcare worker	22	36
	Cancer association	5	8
	Awareness Campaigns	4	7
	Posters	6	10
	Radio	2	3
	School	1	2

### Attitudes of participant women on cervical cancer and Pap smear testing

According to Table 6, majority n = 84 (67%) of the respondents strongly agreed if screening is for free, they will screen. Again, majority strongly agreed that cervical cancer is a serious disease n = 95 (76%). And lastly, a greater portion strongly agreed that that they would screen in the near future. Conversely, n = 6 (5%) neither agreed nor disagreed when asked about screening in the future.

**Table 6:** Attitudes toward cervical cancer and screening amongst participants who are aware of cervical cancer n = 125

		Frequency	%
If screening is free will you screen?*	Strongly disagree	0	0
	Disagree	0	0
	Neither agree nor disagree	14	11
	Agree	27	22
	Strongly agree	84	67
Do you think cervical cancer is a serious disease?*	Strongly disagree	0	0
	Disagree	0	0
	Neither agree nor disagree	5	4
	Agree	25	20
	Strongly agree	95	76
Do you think you will go for screening in the future?*	Strongly disagree	0	0
	Disagree	0	0
	neither agree nor disagree	6	5
	Agree	28	22
	Strongly agree	91	73

*One missing value

### Factors associated with Pap smear uptake

#### Sociodemographic variables associated with screening uptake

From Table 7 above (using the Pearson’s Chi-Square Test), it can be observed the presence of relationships between age and Pap smear screening as well as between residing eras of participants and Pap smear screening.

**Table 7:** Relationship between categorical variables

Pearson chi-square tests		
	Screen for cervical cancer	
Age	Chi-square	14.89
	Sig	.003^*,a,b^
Religion	Chi-square	0.691
	Sig	.406^a,b^
Educ	Chi-square	0.465
	Sig	.793^a,b^
Marital status	Chi-square	0.251
	Sig	0.617
Parity	Chi-square	2.788
	Sig	0.248
Suburb	Chi-square	22.579
	Sig	.020^*,a,b^
Employment	Chi-square	1.643
	Sig	.440^a,b^

Results are based on nonempty rows and columns in each innermost sub table

*The Chi-square statistic is significant at the .05 level

^a^More than 20% of cells in this sub table have expected cell counts less than 5. Chi-square results may be invalid

^b^The minimum expected cell count in this sub table is less than one. Chi-square results may be invalid

## Discussion

### Awareness of cervical cancer, Pap smear purpose and screening uptake

In this study a high level of cervical cancer and Pap smear awareness was recorded across all age groups, with the highest amongst women between the ages 36–60. Considering that this is the age range that carries the highest risk for the development of invasive cervical carcinoma [18], it is good that most knew about the disease. This high awareness level was perhaps attributed to the fact that majority of the women had secondary or higher education. Age group and marital status were the only socio demographic variables that were significantly associated with uptake of Pap smear screening (*p* = 0.003, *p* = 0.02 respectively). Even though a high awareness was reported with tertiary educated women, screening uptake was not positively associated with this group. These findings are in line with a study conducted in Nigeria [19]. Additionally, more people knew the Pap smear test is designed for screening of cervical cancer.

Despite more than half perceiving themselves at risk, only (46.3%) had ever been screened. Others (7.4%) were not certain whether they were at risk. Not knowing whether you are at risk could cause women not to go for screening. Although screening practices stood at 46.3%, this was higher compared to a study done in Kwazulu-natal, South Africa (18%). This huge difference could be attributed to the fact that the South African study was conducted in the rural parts of Kwazulu-natal [20].

### Screening frequency and source of information

Close to half of the women who have had a Pap smear test done, reported going on more than two occasions. Research shows that women in developing countries who have cervical cancer are often the ones who never went for screening or only screened once in their lifetime.

Approximately, 36% stated that they were informed by their healthcare workers, followed by relative/friends 18%. Others stated Pamphlets (16%), Posters (10%). Only 2% stated school being their source of information. However, a study conducted in Ghana revealed school to be the main source of information (37%) [21]. The study was conducted amongst health professionals with tertiary education, which explains why most might have heard about cervical cancer in school. It is important that women become more health literate from a young age, and this can be achieved if cervical cancer and other gynaecological diseases are taught in lower education levels.

The motivations behind screening were mostly done due to recommendation from their health care professionals. This alludes to the fact that women might be of the belief that only through doctor’s recommendation may they go for screening. Other women were motivated to get screened because they knew someone who previously went for screening. This shows that women’s experiences may influence the choice to screen in others. A small proportion were motivated to go for screening as they believed that they might have fibroids. This points to misinformation about the use of Pap smear testing.

The predominant reasons for not screening were that they had not decided on when to go, fear of pain from the procedure, having no time to visit the clinic and no recommendation from a doctor to do so. Similarly, in [22], large number of women in the study were employed, which may explain why some stated that they did not have time to go and visit the doctor. Women who are bread winners in their household may therefore prioritise their work over their health. A study in Malaysia also found that doctor recommendation influenced screening uptake amongst women [23]. Attention needs to be shifted in this area to better equip women to understand the importance of making their health a priority and need to make use of basic services to increase Pap smear testing.

### Perception on risk factors and prevention

Results revealed that most of the women have a poor perception on what is mostly causing women to suffer from invasive cervical cancer. Only 10% of the women indicated having a weak Immune system caused by HIV, along with co infection with HPV carries the highest risk for cervical cancer. It is well documented that progression from low grade lesions to invasive cervical carcinoma is faster amongst HIV positive women. This was due to HPV infection persisting for longer durations in this group [24] It is important that women are educated on HPV and its spread, because it is the causal agent behind majority of the cervical cancer cases.

Other risk factors such as having multiple sexual partners (39.7%), early sexual activity debut (34.9%), smoking (17.5%), and long term oral contraceptive use (15.9%) were mentioned, however it was also poorly reported. Some believed that high blood pressure, irregular menstruation, genetics, and microwaved food caused cervical cancer. There is a need to clear up these misconceptions as to what causes cervical carcinoma, as this indicates that the population at large may share in these beliefs.

### Attitude towards cervical cancer and its screening method

Overall, the participants displayed a positive attitude towards screening, with 73% strongly agreeing to screen in the future. Approximately 73% strongly agreed that cervical cancer is a serious disease, with only 4% neither agreeing nor disagreeing. A positive attitude towards screening may translate into women being more open to the idea of getting tested in the future.

## Conclusion

The results show that women go for screening for various reasons that does not include screening for cervical cancer. Healthcare workers should therefore offer consultations beforehand to find out the reason for women going for Pap smear screening and clear up any misconceptions held about the purpose of a Pap smear test.

A positive attitude was noted amongst participants even though screening practice was low. The results also suggested women come to rely heavily on their health care professionals to recommend screening tests for them. Health care workers must therefore educate women more on their basic health and make cervical cancer screening discussions a part of consultations. Also, very few women knew about HPV vaccination. More talks need to be held on this front along with vaccinations to be given to young girls to reduce cervical cancer incidence later on in life.

It is encouraged that media coverage be improved by using various outlets such as television, social media platforms and radio. Many people have access to radios and television, so this can help to create community awareness. In addition to this, awareness campaigns should focus on spreading awareness about women’s susceptibility and the benefits of regular screening as a low perceived risk can lead to women defaulting on screening.

### Bias and limitation

This study employed Convenience sampling method. Although this method is preferred due to the low cost and time associated with it, it is difficult to generalize the results of the survey to the population it is a non-probability sampling technique. The aim was to enrol 152 women in this study, but due to time constraints and willingness of participants only 136 women took part. With surveys, social desirability bias may be introduced.

## Data Availability

All data generated or analysed during this study are included in this published article.
